# Relation between Pro-inflammatory Cytokines and Acetylcholine Levels in Relapsing-Remitting Multiple Sclerosis Patients

**DOI:** 10.3390/ijms131012656

**Published:** 2012-10-03

**Authors:** Marcella Reale, Federica de Angelis, Marta di Nicola, Elisabetta Capello, Maria di Ioia, Giovanna de Luca, Alessandra Lugaresi, Ada Maria Tata

**Affiliations:** 1 Department of Experimental and Clinical Sciences, University “G. d’Annunzio” Chieti-Pescara, Via Dei Vestini, 31 66100 Chieti, Italy; E-Mail: m.dinicola@unich.it; 2 Department of Biology and Biotechnologies Charles Darwin, Sapienza, University of Rome, Research Center of Neurobiology D. Bovet, P.le Aldo Moro, 5 00185 Roma, Italy; E-Mail: federica.deangelis@uniroma1.it; 3 Department of Neuroscience, Ophthalmology and Genetics, University of Genova, 16126 Genova, Italy; E-Mail: ecapello@neurologia.unige.it; 4 Department of Neuroscience and Imaging, University “G. d’Annunzio” Chieti-Pescara, 66100 Chieti, Italy; E-Mail: a.lugaresi@unich.it (A.L.)

**Keywords:** acetylcholine, pro-inflammatory cytokines, multiple sclerosis

## Abstract

Multiple sclerosis (MS) is a chronic inflammatory, demyelinating and neurodegenerative disorder. Since acetylcholine (ACh) is known to participate in the inflammatory response, we investigated the possible relationship between pro-inflammatory cytokines and acetylcholine levels in relapsing-remitting multiple sclerosis (RR-MS) patients. Levels of ACh and pro-inflammatory cytokines IL1-β and IL-17 were measured both in cerebrospinal fluid (CSF) and sera of 22 RR-MS patients in the relapsing phase and in 17 control subjects affected by other non-neurological diseases (OND). We observed higher levels of pro-inflammatory cytokines such as IL-1β and IL-17 in both CSF and serum of RR-MS patients compared to control subjects. Moreover, ACh levels were lower in CSF and serum of RR-MS patients compared to levels of control subjects. Although the relationship between high inflammatory cytokine levels and low ACh levels need to be further investigated in the future, our data suggest that IL-1β, and cytokines induced by it, such as IL-17 and ACh, may be involved in the pathogenesis of MS.

## 1. Introduction

Multiple sclerosis (MS) is characterized by an altered balance of pro- and anti-inflammatory cytokines that causes a high inflammatory state in the central nervous system (CNS), followed by selective destruction of myelin. Inflammatory cytokines as well as oxidative agents are present in MS lesions [1,2], suggesting that the increased production of these toxic molecules may impair cellular defense mechanisms, facilitating demyelination and counteracting remyelination. IL-17 is one of the inflammatory cytokines secreted mainly by activated T cells and similarly to TNF-α and IL-1, IL-17 has pro-inflammatory properties, playing a key role in the pathogenic mechanisms of MS [3].

Thus, the control of inflammation and neuroprotection should be the key points in designing MS therapeutic protocols. Novel research tools and therapeutic approaches are oriented towards the identification of new molecules that may decrease the level of the noxious agents, and at the same time stimulate oligodendrocyte (OL) progenitors to proliferate and differentiate.

Among the endogenous molecules that may have neuroprotective effects there are the neurotransmitters and, in particular, acetylcholine (ACh). In fact, it has been demonstrated that ACh reduces the inflammatory state as indicated by chronic administration of selective acetylcholinesterase inhibitors (AChEI) in autoimmune encephalomyelitis (EAE) animal models [4]. Several ACh receptor subtypes (muscarinic and nicotinic types) are expressed by OLs and their precursors. As recently demonstrated, the muscarinic agonists are able to induce oligodendrocyte progenitor cell (OPC) proliferation and inhibit the expression of myelin proteins (e.g., myelin basic protein) in mature OLs [5]. On the other hand, the selective stimulation of M2 receptors causes a decrease of OPC progenitor survival, suggesting that the levels of ACh present in the environment and the set of muscarinic receptor subtypes expressed by each subject might play a relevant role in the control of OL progenitor survival, proliferation and differentiation [5].

The involvement of ACh in the modulation of inflammatory states has also been reported. T-cells express both muscarinic (mAChRs) and nicotinic (nAChRs) receptors. Their activation produces differential effects since muscarinic receptors enhance pro-inflammatory mediators, while nicotinic receptor activation exerts an anti-inflammatory response [4,6]. In fact, muscarinic receptor stimulation can elicit upregulation of c-fos, nitric oxide synthase and IL-2 production [6]. On the other hand, ACh, through the α7 nicotinic receptors, can inhibit the release of TNFα and IL-1 from macrophages, suggesting the existence of a “cholinergic anti-inflammatory pathway” [7]. Moreover, it has also been demonstrated that human T cells can synthesize ACh, suggesting that it may be an autocrine signal modulating T cell-dependent immune response [8,9]. Although it is not known whether ACh participates in the immune system activity, it is now evident that lymphocytes and macrophages express an independent cholinergic system [4,8].

In consideration of these data, we investigated the possible involvement of ACh in the inflammatory state of MS patients, with the hypothesis that ACh levels are reduced in active MS thereby leading to heightened inflammation. For this purpose we measured the cytokine production and ACh levels in CSF and sera of Relapsing-Remitting (RR)-MS patients and in control subjects affected by other neurological diseases.

## 2. Results and Discussion

### 2.1. Cytokine Levels

In the group of 22 RR-MS subjects in the relapsing phase, the analysis of cytokines showed that IL-17 levels in serum were significantly elevated compared to control subjects (*p* < 0.05), while in the CSF the levels were lower than in the control group although the differences were not statistically significant (*p* = 0.201). However, in both RR-MS and control group, the mean level of IL-17 was higher in the CSF than in serum (Table 1). Similarly, the level of IL-1β in the CSF and serum from RR-MS patients was significantly higher than in control subjects (*p* < 0.05). In the group of RR-MS subjects, levels of serum IL-1β were about four times higher than CSF levels (*p* = 0.021) (Table 1).

We also measured the levels of IL-10, TNF-α and IL-4 both in CSF and sera and we did not find statistical differences between RR-MS patients and control subjects (data not shown).

### 2.2. Measurement of Acetylcholine Levels

Since ACh takes part in the modulation of inflammation, we also measured the levels of ACh in the CSF and serum of RR-MS patients. ACh levels were significantly lower in RR-MS patients than in control subjects, both in sera and CSF (Table 1). In fact, the ACh levels in CSF of RR-MS patients were 124.3 ± 56.4 pmol ACh/mL compared to 393.6 ± 142.5 pmol ACh/mL measured in control subjects (*p* < 0.05). Similarly, in the serum of RR-MS patients, ACh levels were 175.4 ± 68.9 pmol ACh/mL while in the control subjects they were 586.9 ± 149.4 pmol ACh/mL (*p* < 0.05) (Table 1).

No relationship was found between ACh and gender (*p* = 0.835). Regression analysis did not show any statistically significant relationship between ACh levels and main demographic and clinical characteristics of patients, such as age (*p* = 0.211), EDSS (*p* = 0.471) and disease duration (*p* = 0.582).

## 3. Discussion

MS is considered an inflammatory autoimmune disease of the CNS with a variable clinical course. Patients can present with a rapid progression of the disease or remain clinically stable for several decades. The qualitative and quantitative levels of cytokines reflect the inflammatory state of the patient and may affect the disease outcome [10]. Peripheral blood and endothelial cells are well known sources of cytokines, but also brain cytokine over-production, deriving from the CNS, might in fact contribute to the peripheral cytokine pool. On the other hand, peripheral cytokines might affect human brain functions by crossing the blood-brain barrier (BBB) and interacting with the CNS [11]. It is known that an acute inflammatory stimulus (such as IL-1 or endotoxin) in the brain, efficiently induces inflammatory cytokine production in the periphery [12,13]. Thus, it seems that the CNS not only is able to produce cytokines, but it may also contribute to increase the peripheral pool. Since cytokines are involved in the regulation of inflammatory responses, their levels may reflect the disease process.

Several studies have analyzed peripheral inflammatory parameters, including cytokines and related molecules in the blood (serum, plasma) of MS patients [14]. The same parameters have been measured less frequently in the CSF. For these reasons we have evaluated the levels of different pro- and anti-inflammatory cytokines both in serum and in CSF of RR-MS patients. Our results confirm the presence, in RR-MS patients, of an altered profile of cytokines typically associated with a strong inflammatory response with high levels of IL-17 in serum and high levels of IL-1β both in serum and in CSF. Comparison of cytokine levels showed higher serum concentrations of the cytokines IL-1β and higher levels of IL-17 in CSF of RR-MS patients. Further studies are needed to establish whether higher levels in the CSF compared to serum of IL-17 could be due to migration of Th17 cells into the CNS, thereby inducing inflammation.

Our data may point out the peripheral/central and inflammatory/regulatory balance which may explain how the timely control of inflammation in the CNS may be a way of limiting peripheral inflammation.

Moreover, several studies have documented the accumulation of Th17 cells in MS lesions, suggesting that Th17 cells may play a central role in the immunopathogenesis of MS [15,16].

**Figure 1:**
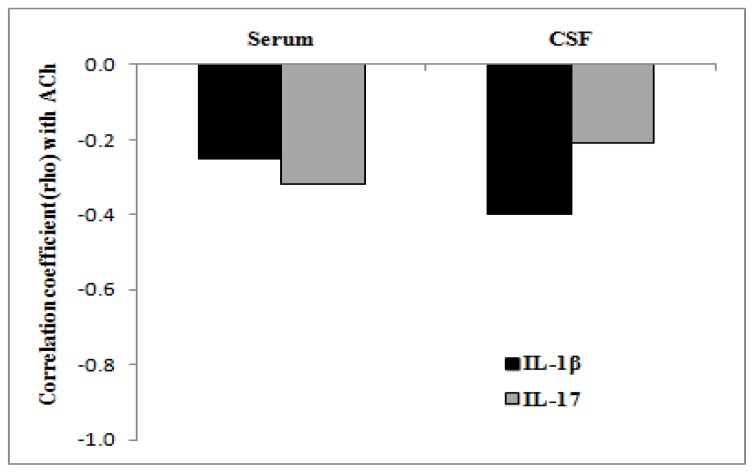
Correlation coefficient between ACh and cytokine levels in serum and CFS of relapsing-remitting multiple sclerosis (RR-MS) patients.

It is known that ACh modulates the production and release of pro- and anti-inflammatory cytokines [3]. Recently, it has also been demonstrated that *in vivo* treatment with AChE inhibitors (e.g., donepezil) down-regulates the expression and production of IL-1β, IL-6 and TNFα and up-regulates the expression and production of IL-4 in peripheral blood mononuclear cells (PBMC) of Alzheimer’s patients [17,18]. Moreover, the AChE inhibitors cause a consistent reduction of CNS lymphocyte infiltrates in autoimmune encephalomyelitis (EAE) animal models ameliorating clinical symptoms [3]. On the other hand, the nicotinic stimulation appears involved in suppression of Th1 and Th17 response [19] and EAE mice, KO for nicotinic receptor α7, show a significant reduction of the number of T lymphocytes migrating to the CNS [20]. These data suggest that ACh controls, probably through different acetylcholine receptor subtypes, the cytokine network possibly by acting on the lymphocyte activity and migration [4,6,20]. Given the effects of ACh on cytokine levels, we have measured the levels of ACh in serum and CSF of RR-MS patients. The fluorimetric assay used in the present work, although less sensitive than HPLC method [21], has already been used to measure ACh levels in various biological fluids without pretreatment or purification of samples [22]. This technique has shown significant differences of concentrations in CSF and serum among RR-MS patients and control subjects. In fact the data obtained indicate the presence of lower levels of ACh both in serum and in CSF of RR-MS patients compared to subjects affected by other neurological diseases. The decreased ACh level may be, at least in the central nervous system, in part dependent on neuron loss that occurs in MS as a consequence of the myelin and axon degeneration. On the other hand, although we did not find any statistically significant correlation between the low levels of ACh and high levels of cytokines both in serum and CSF (see Figure 1), the decreased levels of ACh in serum of RR-MS patients suggest that ACh levels may be correlated with the high inflammatory state.

Our study, for the first time, investigated ACh levels in biological fluids of RR-MS patients. Interestingly the levels of ACh we found in CSF and serum of RR-MS patients were significantly lower than levels detected in OND (control) patients and significantly different compared to the levels reported in other studies, e.g. in patients treated with analgesic drugs after acoustic neuroma resection [21] or affected by other neurological diseases (e.g., Parkinson’s disease, Alzheimer’s disease and amyotrophic lateral sclerosis) [23]. We hypothesize that low levels of ACh may correlate with diseases classically characterized by an impairment of ACh neurotransmission, or the treatment with analgesic drugs may directly or indirectly affect the levels of ACh that usually participate in the modulation of nociception stimuli [24]. Finally the different methods used to measure ACh levels [21,22–24] might hamper the comparison between studies on subjects affected by different neurological diseases.

## 4. Experimental Section

### 4.1. Patients

Patients with MS diagnosed according to the revised McDonald criteria [25] and followed by the MS Center of the University “G. d’Annunzio” of Chieti and by the Department of Neuroscience, University of Genova (Italy) were included in the study. The diagnosis of RR-MS was confirmed by brain magnetic resonance imaging (MRI) with gadolinium, and twenty-two RR-MS patients with active disease were enrolled in the present study. In this study, all patients were judged to have active disease according to clinical criteria, that means the appearance of new symptoms or a significant aggravation of pre-existing symptoms within the last 1 months. Subjects with other neurological diseases (OND, *n* = 17; patients with chronic back pain (*n* = 6), chronic tension headache (*n* = 5), ischemic transverse myelopathy (*n* = 6); were also included in the study as controls. All subjects gave their informed consent in following with the declaration of Helsinki. None of the patients had received corticosteroids or immunosuppressive drugs in the 6 months prior to blood withdrawal. None of the subjects had any inflammatory diseases, in the previous month, that may have been associated with increases in cytokine secretion.

Phlebotomy and lumbar puncture were performed in the same morning between 08:00 and 10:00. Serum obtained from all subjects was centrifuged and aliquots were stored at −80 °C until cytokine assays were performed. Lumbar puncture was performed for diagnostic purposes in all cases, and the CSF collected was centrifuged at 2000*g* for 10 min at 4 °C, aliquoted and were snap-frozen within 20 min after harvesting and stored at −80 °C until analysis. A consecutive code number was assigned to each sample to ensure that all assays were performed in a blinded manner. All samples were analyzed not more than sixth months after collection. The main characteristics of the patients involved in the study are summarized in Table 2.

### 4.2. Cytokine and Acetylcholine Level Measurement

All cytokines were measured using commercial ELISA (Endogen, USA) kits, in accordance with manufacturer’s instructions. All steps were performed in duplicate and at room temperature. Cytokine levels were then calculated plotting the optical density (O.D.) of each sample against the standard curve. Duplicate values that differed from the mean by greater than 10% were not considered for further analysis.

ACh was measured by commercial colorimetric/fluorimetric kit (Abcam, Cambridge, UK) [22]. Fifty microliter of the sample (CSF or serum) was mixed with 50 μL of reaction solution including choline assay buffer, choline probe, enzyme mix and AChE according to the instructions. The level of Ch/ACh (pmol/well) was calculated by plotting the fluorescence of each sample in relation to choline standard curve. The standard curve, according to the fluorimetric procedure as indicated by manufacturer’s instructions, was obtained by diluting the Choline Standard to generate 0, 10, 20, 30, 40, 50, 100 and 200 pmol/well of the Choline Standard. The measurement of the fluorescence was obtained by Glomax Multi Detection System (Promega) at λ Ex/Em 535/587 nm. Considering the intensity of the fluorescence of the samples, the *x* values (corresponding to Ch/ACh concentration and reported as pmol/well) were calculated by the standard curve equation using GraphPad Prism 5.0. The limit of detection of Choline is defined by the analyte concentration resulting in fluorescence higher than that of the dilution medium (0 dose of standard choline = Blank).

### 4.3. Statistical Methods

The qualitative variables were summarized as frequency and percentage and quantitative variables as median and range or mean and standard error. The results were reported separately for RR-MS and control groups. Differences in characteristics of the patients and cytokine/ACh levels among RR-MS and control groups were tested by Mann-Whitney U test and Fisher’s exact test for continuous and categorical variables, respectively. Differences between serum and CSF values were tested by Wilcoxon U test.

To evaluate the relationship between ACh and main demographic and clinical characteristics of patients we performed univariate regression analysis or Mann-Whitney U test for quantitative and qualitative variables, respectively. The Spearman rho correlation coefficient was applied to evaluate the correlation between ACh and cytokine levels. Statistical analysis was performed using SPSS^®^ Advanced Statistical 11.0 software (SPSS Inc, Chicago, Illinois, USA).

## 5. Conclusions

The cholinergic anti-inflammatory pathway is a physiological neuro-immune mechanism that regulates innate immune function and controls inflammation inhibiting the production of pro-inflammatory cytokines and chemokines and suppressing the activation of nuclear factor-kappa B expression and oxidative system [26,27]. Although in our RR-MS patients the no significant correlation between cytokine and ACh levels was found (Figure 1), probably due to the relatively low sample number, it is intriguing that ACh and pro-inflammatory cytokine levels varied in an inverse manner. Considering the ability of ACh to modulate inflammatory responses and OPC proliferation, the decreased levels of ACh observed in RR-MS patients may contribute to maintenance of an inflammatory state and exacerbate the symptoms of MS. Although further analysis will be necessary to elucidate the role of the cholinergic pathway in the pathogenesis of MS, our observations together with the evidence that AChE inhibitors ameliorate the clinical symptoms in EAE models [4] point out a new possible role for ACh in MS.

## Figures and Tables

**Table 1:** Cytokine and acetylcholine (ACh) levels in serum and cerebrospinal fluid (CSF) (mean ± standard error).

Variable	Control group (*n* = 17)		Wilcoxon U test *p*-value	RR-MS group (*n* = 22)		Wilcoxon U test *p*-value
	
	Serum	CSF		Serum	CSF	
IL-17 (pg/mL)	8.3 ± 2.7	25.7 ± 2.9	0.144	15.7 ± 2.4*	20.6 ± 2.9	0.208
IL-1β (pg/ mL)	19.4 ± 4.3	6.2 ± 0.8	0.013	97.4 ± 26.1*	25.0 ± 7.2*	0.021
ACh (pmol/ mL)	586.9 ± 149.4	393.6 ± 142.5	0.917	175.4 ± 68.9*	124.3 ± 56.4*	0.686

Wilcoxon U test *p*-value relative to comparison serum *vs.* CSF values within each group;

* *p* < 0.05;

Mann-Whitney U test relative to comparison RR-MS group *vs.* Control group

**Table 2:** Characteristics of the patients involved in this study.

Variable	Control group (*n* = 17)	RR-MS group (*n* = 22)	*p*-value
Gender, *n (%)*			0.275^a^
Male	2 (11.8)	7 (31.8)	
Female	15 (88.2)	15 (68.2)	
Age (years), *median (range)*	41 (19–76)	37 (18–59)	0.377^b^
BBB impairment, *median (range)*	5.1 (2.4–17.3)	5.5 (2.8–15.2)	0.986^b^
Duration of disease (years), *median (range)*	4 (3–6)	5 (3–6)	0.975^b^
EDSS, *median (range)*	-	2.5 (0.0–6.0)	

BBB: Blood-Brain Barrier; EDSS: Expanded Disability Status Scale;

a Fisher’s exact test;

b Mann-Whitney U test.
